# Trim13‐induced ubiquitination of RPS27A inhibits the progression of lung cancer by depending on the inactivation of NF‐κB signaling pathway

**DOI:** 10.14814/phy2.70157

**Published:** 2024-12-12

**Authors:** Lailing Li, Hui Zhou, Yayun Cui, Ke Xu

**Affiliations:** ^1^ Division of Life Sciences and Medicine, Department of Respiratory Medicine, the First Affiliated Hospital of USTC University of Science and Technology of China (Anhui Provincial Cancer Hospital) Hefei Anhui China; ^2^ Division of Life Sciences and Medicine, Department of Cancer Radiotherapy, the First Affiliated Hospital of USTC University of Science and Technology of China (Anhui Provincial Cancer Hospital) Hefei Anhui China

**Keywords:** lung cancer, NF‐κB signaling, RPS27A, TRIM13, ubiquitination

## Abstract

Lung cancer (LC) is the leading cause of cancer‐related death worldwide. Recent studies have shown that tripartite motif 13 (TRIM13) play important regulatory roles in the progression of different tumors. In this study, we focused on the role of TRIM13 in LC tumorigenesis and its underlying molecular mechanisms. The study demonstrated TRIM13 was identified as a novel tumor suppressor gene of LC and its overexpression suppressed LC progression in vitro and in vivo. Mechanistically, TRIM13 interacted with RPS27A, increasing RPS27A ubiquitination and degradation. Furthermore, RPS27A overexpression reversed the inhibitory effect of TRIM13 overexpression on LC progression. By binding to RPS27A and encouraging its ubiquitination and degradation, TRIM13 hindered LC advancement. We also found that RPS27A overexpression reversed the inhibitory effect of TRIM13 overexpression on NF‐κB signaling, thereby further promoting the proliferation and metastasis of LC cell lines. Therefore, targeting the TRIM13/RPS27A/NF‐κB signaling axis may be a promising target for LC treatment.

## 1 INTRODUCTION

Lung cancer (LC) is a deadly malignancy and the second leading cause of cancer‐related deaths worldwide (Sung et al., 2021). The rapid development of surgical resection, chemotherapy, radiotherapy and immunotherapy in recent years has significantly improved the treatment of LC patients (Hirsch et al., 2017; Steven et al., 2016). However, due to the lack of typical early symptoms, more than 62% of patients are diagnosed with advanced LC, and their five‐year survival rate is still less than 25% (Jones & Baldwin, 2018; Schabath & Cote, 2019). In addition, the high recurrence rate and distant metastasis also impose a heavy health burden on LC patients, especially those with advanced LC (Fedor et al., 2013; Li, Liu, et al., 2022; Slim et al., 2021). Recently, gene therapy, which involves modifying cells (either in vitro or in vivo) using genetic material to assist in achieving disease cures, has shown promising applications in the field of cancer (Mulligan, 1993). Significant therapeutic potential has been demonstrated through a variety of gene therapy drugs tested in numerous in vitro and preclinical animal models. For instance, in a melanoma model, injecting the adenovirus carrying the cancer cell death inducer MDA‐7 into tumors of 28 patients resulted in complete remission in two patients and partial remission in others (Cunningham et al., 2005). Systemic immune activation and local cell apoptosis were observed in another 22 patients (Tong et al., 2005). In a large Phase I study involving histologically confirmed advanced cancer patients, delivering the tumor necrosis factor TNF‐α using the adenoviral vector TNFerade into patient tumors led to objective responses in 43% of patients, with 5 out of 30 patients showing complete response to treatment (Senzer et al., 2004). Overall, gene therapy holds great promise in enhancing current cancer treatment outcomes. Therefore, there is an urgent need to explore the progression and metastasis mechanisms of LC and to identify new diagnostic and therapeutic biomarkers to provide positive and effective strategies for the treatment of LC patients.

The tripartite motif (TRIM) family is one of the subfamilies of single‐protein ring finger E3 ubiquitin ligases, with more than 80 protein members (Huang et al., 2022; Reymond et al., 2001). Accumulated research evidence shows that members of the TRIM family are involved in diverse cell signal transduction and biological processes, including autophagy (Kumar et al., 2017), cell cycle progression (Venuto & Merla, 2019), immune activation and inflammatory processes (Di Rienzo et al., 2020), apoptosis (Mandell et al., 2020), and DNA damage response (McAvera & Crawford, 2020), etc. Given the above regulatory functions, different TRIM family members exert multiple effects on cancer cell phenotype, proliferation, migration and invasion in cancer. For example, TRIM29 silencing reduced Wnt/β‐catenin signaling pathway activity in LC cells, inhibiting cell proliferation and colony formation, suggesting that TRIM29 is an oncogene that promotes LC progression (Qiu et al., 2015). Up‐regulation of TRIM22, which is down‐regulated in osteosarcoma, plays a tumor‐suppressive role by reducing the proliferation and metastasis of osteosarcoma cells (Liu et al., 2022). TRIM is an important factor in the progression of cancer development and metastasis.

TRIM13 (also known as RFP2, DLEU5), which is located within the most frequently deleted region on chromosome 13 (13q14), has been shown to be deleted in a variety of malignancies (Crawford et al., 2018; Yu et al., 2023). For example, as reported by Chen et al., TRIM13 was downregulated in esophageal cancer, ovarian cancer, leukemia, especially breast cancer, and lower TRIM13 expression levels were associated with poorer distant metastasis and recurrence‐free survival in breast cancer patients. It suggested that TRIM13 is a promising marker for breast cancer prognosis (Chen et al., 2019). Furthermore, Gatt et al. demonstrated that downregulation of TRIM13 in multiple myeloma (MM) reduced the cell cycle progression and proliferation of tumor cells by inhibiting the activity of the NF kappa B pathway and the 20S proteasome. TRIM13 has great potential as a new target for MM therapeutic intervention (Gatt et al., 2013). The above findings suggest that TRIM13 not only acts as a tumor suppressor but also as a potential predictive biomarker for cancer prognosis. However, the potential function and mechanism of action of TRIM13 in LC are still unclear.

In this study, we found that TRIM13 was lowly expressed in LC tissues and cell lines, which were associated with lower overall survival (OS). Subsequently, we performed cell experiments and revealed that TRIM13 overexpression suppressed the proliferation and metastasis of LC cells. RPS27A overexpression reversed the inhibitory effect of TRIM13 overexpression on NF‐κB signaling, thereby further promoting the proliferation and metastasis of LC cell lines. Finally, we performed a mechanistic study and showed that TRIM13 overexpression suppressed LC growth and metastasis by attenuating the expression level of RPS27A. This study revealed a novel role and underlying mechanism of the NF‐κB signaling axis in LC progression. Therefore, TRIM13/RPS27A/NF‐κB signaling axis may be a promising target for the treatment of LC.

## 2 MATERIALS AND METHODS

### 2.1 Tissue samples and cells

LC tumor tissues and paired normal tissues were collected during the surgery of patients. The obtained tissue samples were quickly frozen with liquid nitrogen and stored at −80°C until subsequent experiments. This study has obtained written informed consent from all enrolled patients and approval from the Ethics Committee of our hospital.

Human LC cell lines (A549 and H157 cells) and human normal lung epithelial cells BEAS‐2B were provided by the Cell Bank of the Chinese Academy of Sciences (Shanghai, China). RPMI‐1640 medium for culturing cells was added with 10% fetal bovine serum (FBS, Gibco, USA) and 1% penicillin and streptomycin (Gibco, USA). Cells were cultured at 37°C and 5% CO_2_.

### 2.2 qRT‐PCR

The total RNA in cells and tissues was extracted by TRIzol reagent (15596026CN, Invitrogen, USA), and then reversed transcribed into cDNA using PrimeScript RT Master Mix Kit (RR036A, TaKaRa, Japan). GAPDH was used as an internal control. Then, qRT‐PCR was performed with the use of the SYBR green PCR kit (DRR041A, TaKaRa, Japan). The mRNA expression levels of genes were calculated by the 2^−ΔΔCt^ method. Primers used in this study are listed in Table 1.

**TABLE 1:** Sequences of all primers.

Gene	Primers sequences (5′‐3′)
TRIM13	F: GTTTTGCCTTGCTCCCACAAC R: TCCTTACGGCATGTAGGACAC
RPS27A	F: AGAAGAAGTCTTAC ACCACTCCC R: TGCCATAAACACCC CAGC
GAPDH	F: CTGGGCTACACTGAGCACC R: AAGTGGTCGTTGAGGGCAATG

### 2.3 Western blot

The total protein in tissues and cells was extracted by RIPA lysate, and then was quantified by BCA protein detection kit (Bio‐Rad, USA). Proteins were separated by 12% SDS‐polyacrylamide gel and transferred to nitrocellulose membrane. The membrane was then blocked in 10% skim milk for 1 h at room temperature and incubated overnight at 4°C with primary antibodies. Subsequently, the membrane was incubated with the corresponding secondary antibody for 2 h at room temperature. GAPDH was used as a protein loading control. Finally, protein bands were detected by chemiluminescent reagents (Sigma‐Aldrich, USA). The primary antibodies employed in western blot analysis included: anti‐KCNRG (1:1000; ProSci, PSI‐28‐315), anti‐PSENEN (1:1000; Abcam, ab154830), anti‐RPS27A (1:1000; Abcam, ab172293), anti‐SLC25A16 (1:2000; ProSci, PSI‐25‐930), anti‐SPRYD7 (1:1000; Invitrogen, MA5‐22347), anti‐UBA52 (1:1000; Abcam, ab109227), anti‐TRIM13 (1:1000; Abcam, ab234847), anti‐HA (1:1000; Abcam, ab236632), anti‐p‐IKKβ (1:1000; Abcam, ab194528), anti‐IKKβ (1:1000; Abcam, ab124957), anti‐MMP9 (1:1000; Abcam, ab76003), anti‐caspase3 (1:5000; Abcam, ab32351), anti‐BCL2 (1:2000; Abcam, ab184925), and anti‐GAPDH (1:1000; Abcam, ab8245). The corresponding secondary antibodies employed in western blot analysis included: goat anti‐rabbit IgG H&L (Abcam, ab6721) and goat anti‐mouse IgG H&L (Abcam, ab205719).

### 2.4 Cell transfection

Ubiquitin‐HA, TRIM13 overexpression plasmid (oe‐TRIM13), RPS27A (oe‐RPS27A) overexpression plasmid and their corresponding controls (oe‐NC) were synthesized by Guangzhou RiboBio Company (Guangzhou, China). According to the manufacturer's instructions, Ubiquitin‐HA, oe‐TRIM13 and oe‐RPS27A were transfected into A549 and H157 cells using transfection reagent Lipofectamine 3000 (Invitrogen, USA). After 48 h of transfection, the cells were collected for subsequent experiments.

### 2.5 Cell proliferation assay

Cell proliferation ability was assessed by Cell Counting Kit‐8 (CCK‐8, C0037, Beyotime, China). LC cells were seeded into 96‐well plates at a density of 5000 cells/well and incubated overnight. Then, at 0, 24, 48 and 72 h of cell transfection, 100 μL of CCK‐8 reagent was added to each well and incubated at room temperature for 1 h. The optical density (OD) at 450 nm of each well was determined by a microplate reader.

### 2.6 Migration and invasion assays

Transwell assays were performed to assess cell migration and invasion abilities. Cell migration is the movement of cells from one place to another in the absence of physical barriers in response to chemical signals (such as chemokines) or other stimuli. Therefore, in Transwell migration experiments, transfected LC cells (3 × 10^4^ cells/200 μL serum‐free medium) were seeded in the upper chamber of the Transwell chamber (8.0 μm pore size, CLS3422, Corning, USA), while the lower chamber contains 800 μL of RPMI‐1640 medium supplemented with 10% FBS. Cell invasion refers to the ability of cells to penetrate the matrix and membrane by secreting enzymes (such as matrix metalloproteinases, MMPs) to degrade the matrix in the presence of physical obstacles (such as extracellular matrix). Therefore, in the Transwell invasion experiment, a layer of Matrigel (354,234, Corning, USA) is laid on the porous membrane to simulate the environment of the extracellular matrix in the body. Then, transfected LC cells (3 × 10^4^ cells/200 μL serum‐free medium) were seeded in the upper chamber of the Transwell chamber, while the lower chamber contains 800 μL of RPMI‐1640 medium supplemented with 10% FBS. Subsequent experimental procedures for cell migration and invasion assays are the same. After incubation for 24 h, the cells on the surface of the lower chamber were fixed with 4% paraformaldehyde and stained with 0.1% crystal violet solution. Stained cells in 6 randomly selected fields of view were counted by an optical microscope.

### 2.7 Co‐immunoprecipitation (Co‐IP) analysis

Total protein in LC cells was extracted with RIPA lysate containing protease inhibitors (P0013E, Beyotime, China). Proteins were quantified by the BCA protein assay kit (23,227, Invitrogen, USA). IgG (P2171, Beyotime, China) or IP antibody, IP matrix and PBS were incubated at 4°C on a rotator for 2 h. Then, the pellet from the mixture obtained by centrifugation was added to the protease inhibitors, and the cell lysates were transferred to the matrix and incubated overnight at 4°C on a rotator. Subsequently, the matrix was centrifuged to obtain immunoprecipitation, and SDS‐PAGE sample loading buffer (P0015L, Beyotime, China) was added, followed by boiling at 100°C for 10 min. IP and input proteins were detected by western blot.

### 2.8 Immunoprecipitation (IP) analysis

IP and western blot assays were conducted to assess the ubiquitination levels of proteins in LC cells. For IP, LC cells were initially lysed with immunoprecipitation lysis buffer. Following quantification, the protein samples were incubated overnight at 4°C with anti‐RPS27A antibodies and protein A/G beads (Thermo, USA). The immunoprecipitates were washed five times with immunoprecipitation washing buffer, after which the immunoprecipitates on the beads were collected via centrifugation and subsequently analyzed by western blot.

### 2.9 Animal experiment

We established a LC xenograft tumor model by subcutaneously injecting A549 cells (2 × 10^5^ cells/100 μL) into the right flank of BALB/c nude mice (4–6 weeks old, male). After 1 week, the mice were randomly divided into three groups and injected via tail vein twice a week: (1) control adenovirus; (2) TRIM13‐overexpressing adenovirus and control adenovirus; (3) TRIM13‐overexpressing adenovirus and RPS27A overexpressing adenoviruses. The maximum (a) and minimum length (b) of mouse tumors were measured every week by calipers, and mouse tumor volume (V) was calculated by the following formula: *V* = 1/2 × ab^2^. After 4 weeks of injection, mice were euthanized by inhalation of mobile CO_2_. Tumors were collected for photographing and weighing. Animal experiments were approved by the Animal Care and Use Committee of the hospital.

### 2.10 Statistical analysis

Prism 9.0 was used to analyze all the data, which were given as mean ± standard deviation from at least three repeated assays. Student *t*‐test and one‐way ANOVA were performed to examine statistical significance. *p* < 0.05 was considered to indicate a statistically significant difference. Overall survival (OS) of LC patients was analyzed by Kaplan–Meier analysis.

## 3 RESULTS

### 3.1 TRIM13 was lowly expressed in LC and connected to a lower overall survival

**FIGURE 1:**
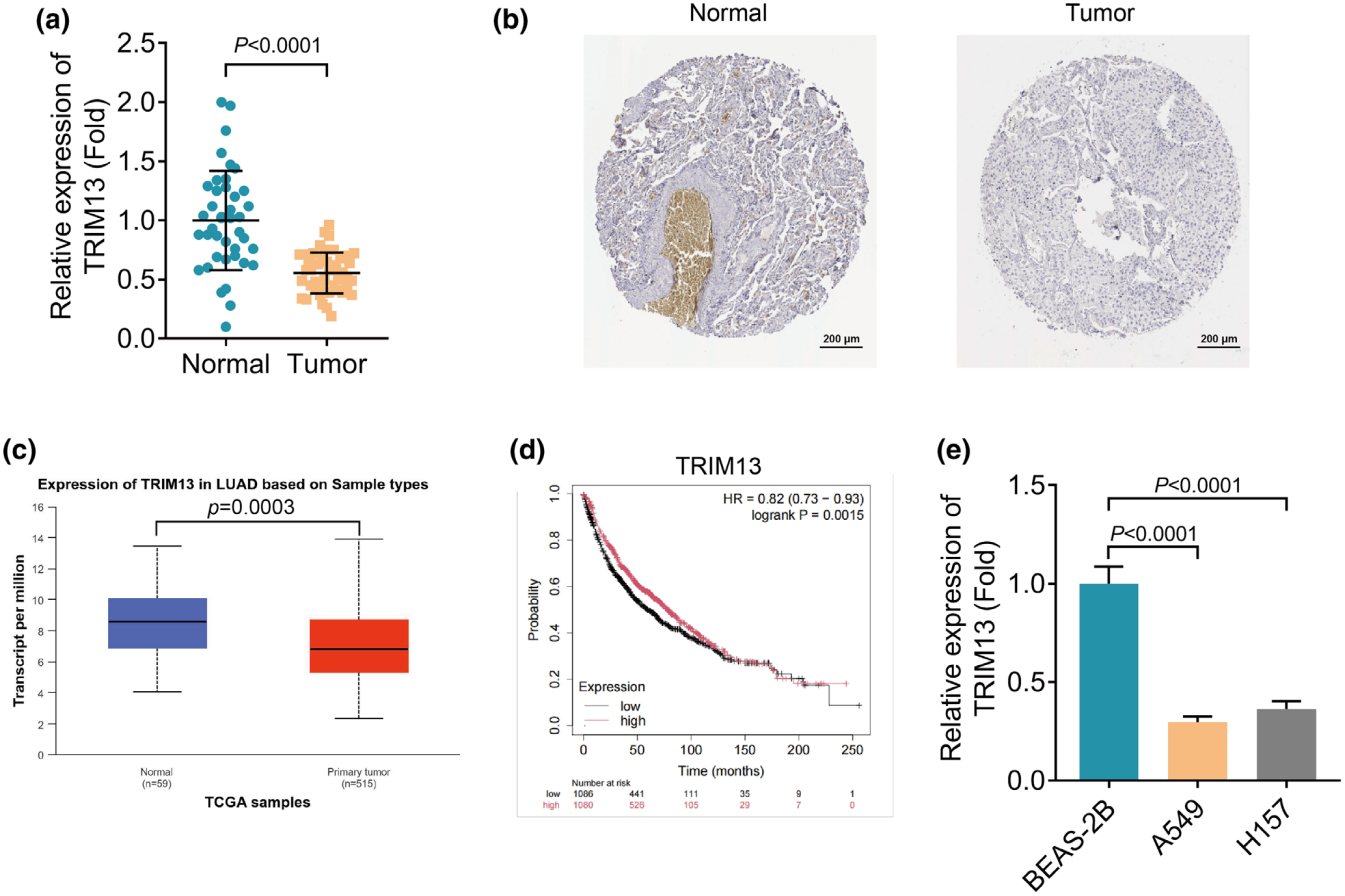
TRIM13 was lowly expressed in LC and connected to a lower overall survival (OS). (a) The mRNA expression level of TRIM13 in LC tumor tissues and adjacent normal tissues was evaluated by qRT‐PCR; (b) IHC analysis of TRIM13 in LC tumor tissues and adjacent normal tissues; (c) The mRNA expression level of TRIM13 in LC samples was analyzed by UALCAN; (d) Kaplan–Meier analysis of TRIM13 expression for the OS of LC; (e) The mRNA expression level of TRIM13 in BEAS‐2B cell line and LC cell lines was evaluated by qRT‐PCR.

We first detected the mRNA expression level of TRIM13 in LC tumor tissues and adjacent normal tissues by qRT‐PCR. Analysis of the data revealed that TRIM13 was significantly downregulated in LC tumor tissues compared with normal tissues (Figure 1a). Besides, immunohistochemistry (IHC) analysis also confirmed the downregulation of TRIM13 in LC tumor tissues (Figure 1b). In addition, an in‐depth analysis of 574 lung adenocarcinoma (LUAD) samples in the TCGA database using the UALCAN website (https://ualcan.path.uab.edu/index.html) showed that TRIM13 mRNA expression was significantly reduced in LUAD tumor tissue samples (Figure 1c). Kaplan–Meier analysis proved that LC patients with low TRIM13 had lower OS (Figure 1d). Moreover, the mRNA expression of TRIM13 in human normal lung epithelial cells BEAS‐2B and LC cell lines (A549 and H157 cells) was determined by qRT‐PCR. Consistently, we found low mRNA expression of TRIM13 in LC cell lines (Figure 1e). Therefore, the above results suggested that TRIM13 was underexpressed in LC and correlated with lower OS of LC patients.

### 3.2 Overexpression of TRIM13 inhibited the proliferation and metastasis of LC cells

**FIGURE 2:**
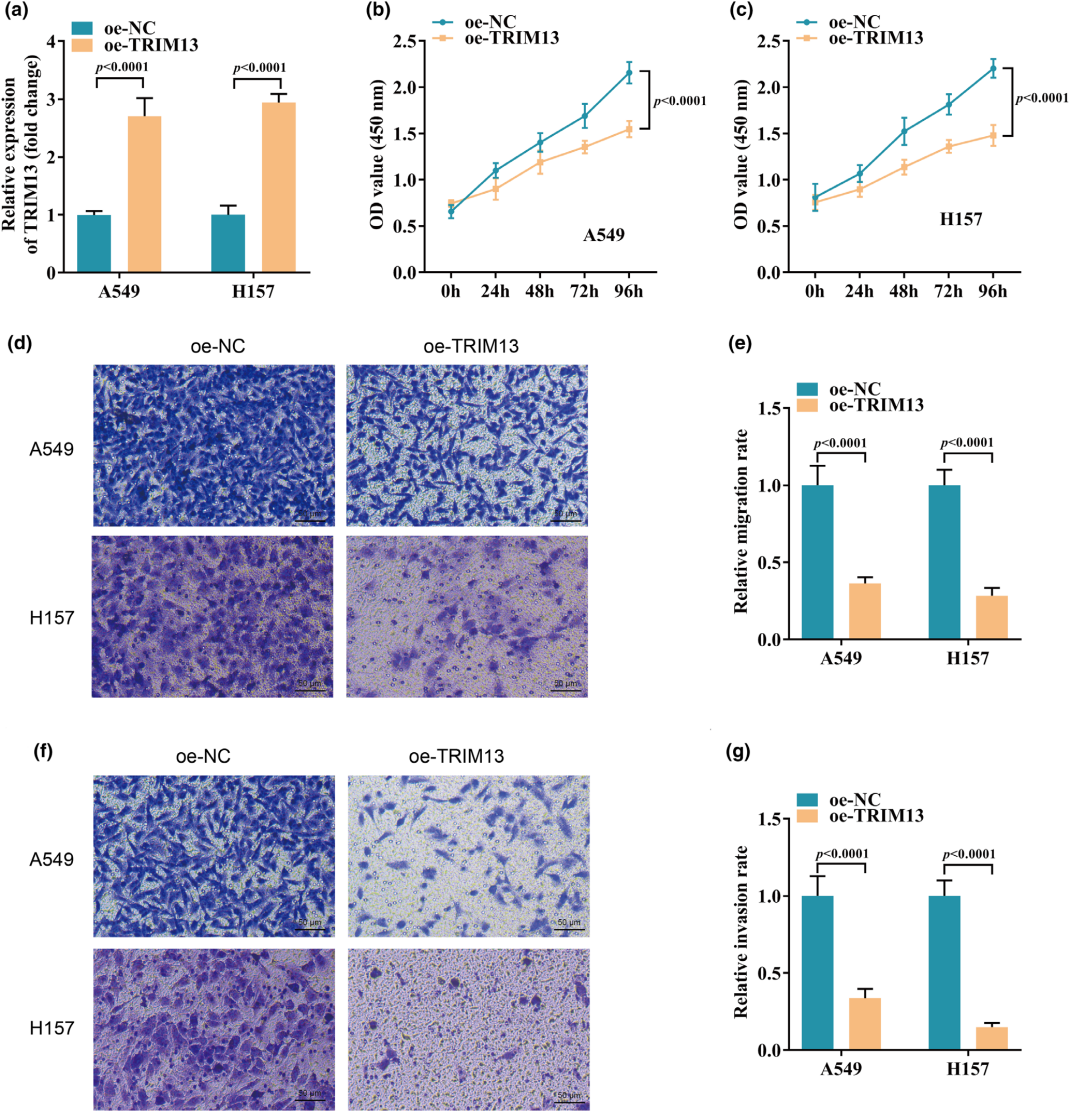
Overexpression of TRIM13 inhibited the proliferation and metastasis of LC cells. (a) The transfection efficiency of TRIM13 in LC cells; (b, c) Cell proliferation was evaluated by CCK‐8 assay; (d–g) Cell migration (d, e) and invasion (f, g) were detected by the transwell assay.

In order to explore the regulatory role of TRIM13 in LC, we conducted a series of cell experiments. We first overexpressed TRIM13 in LC cell lines A549 and H157 by cell transfection. Subsequently, we detected the transfection efficiency by qRT‐PCR, and the results showed that oe‐TRIM13 transfection significantly up‐regulated TRIM13 in A549 and H157 cells (Figure 2a). The results of CCK‐8 assay indicated that the cell proliferation of A549 and H157 was greatly inhibited after TRIM13 overexpression (Figure 2b,c). In addition, transwell analysis revealed that upregulation of TRIM13 in A549 and H157 cells inhibited cell migration (Figure 2d,e) and invasion (Figure 2f,g). These results recommended that TRIM13 overexpression may inhibit LC progression by weakening tumor cell proliferation and metastasis.

### 3.3 TRIM13 interacted with RPS27A and enhanced RPS27A ubiquitination and degradation to downregulate RPS27A expression

**FIGURE 3:**
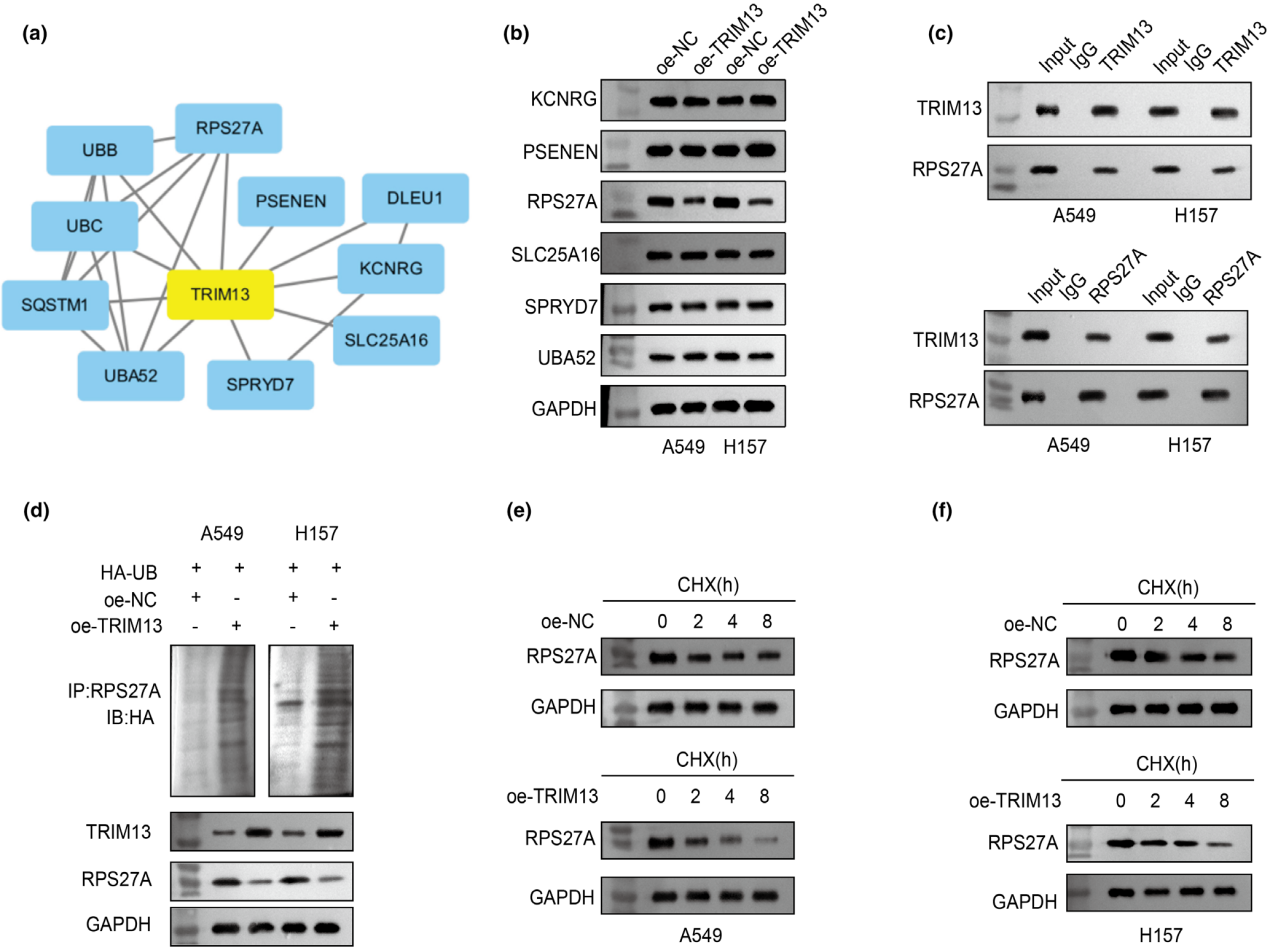
TRIM13 interacted with RPS27A and enhanced RPS27A ubiquitination and degradation to downregulate RPS27A expression. (a) The proteins that interacted with TRIM13 predicted by GEO database; (b) The expression levels of six predicted interacting proteins (UBA52, SPRY7, SLC25A16, RPS27A, PSENEN and KCNRG) in TRIM13 overexpressed LC cells were evaluated by western blot; (c) Exogenous protein interactions between TRIM13 and RPS27A in LC cells were detected by the Co‐IP analysis; (d) The amount of ubiquitin co‐immunoprecipitated with RPS27A in TRIM13 overexpressed LC cells was evaluated by western blot; (e, f) In the presence of CHX (2 μg/mL), the RPS27A protein levels in LC cells transfected with oe‐NC and oe‐TRIM13 was detected at 0, 2, 4, and 8 h by western blot.

To further investigate the potential mechanism by which TRIM13 regulates LC proliferation and metastasis, we predicted the proteins that interacted with TRIM13 through the GEO database. The protein interaction network diagram revealed 10 candidate proteins, that was Ubiquitin A‐52 residue ribosomal protein fusion product 1 (UBA52), SPRY domain‐containing protein 7 (SPRY7), SLC25A16, ribosomal protein S27A (RPS27A), PSENEN, KCNRG, DLEU1, ubiquitin B gene (UBB), ubiquitin‐conjugating enzyme (UBC) and SQSTM1 (Figure 3a). To identify TRIM13‐interacting proteins, we first detected the expression levels of six predicted proteins (UBA52, SPRY7, SLC25A16, RPS27A, PSENEN and KCNRG) in TRIM13‐overexpressed A549 and H157 cells. The results of western blot analysis showed that TRIM13 overexpression significantly inhibited the expression level of RPS27A, but had no effect on the expression levels of the other five proteins in A549 and H157 cells (Figure 3b). Therefore, RPS27A was identified as a protein interacting with TRIM13. Subsequently, Co‐IP analysis was performed in LC cells to confirm the interaction of RPS27A with TRIM13. As shown in Figure 3c, TRIM13 was precipitated by RPS27A in LC cells. The above findings supported the interaction between TRIM13 and RPS27A.

Afterwards, we explored the molecular mechanism by which TRIM13 regulates the expression level of RPS27A protein. Given that TRIM13 functions as an E3 ubiquitin ligase (Crawford et al., 2018) and RPS27A may undergo ubiquitination, we hypothesized that TRIM13 may downregulate RPS27A expression by promoting RPS27A protein ubiquitination. To prove this assumption, we performed IP and westren blot assays. As shown in Figure 3d, the ubiquitization level of RPS27A was significantly increased in TRIM13‐overexpressed LC cells. Then, we subjected LC cells to the protein synthesis inhibitor cycloheximide (CHX) to explore the impact of TRIM13 overexpression on the stability of endogenous RPS27A protein. Findings demonstrated that TRIM13 upregulation greatly accelerated RPS27A degradation (Figure 3e,f). The above results indicated that TRIM13 enhanced RPS27A protein ubiquitination and degradation, which in turn downregulated RPS27A expression.

### 3.4 Overexpression of RPS27A significantly reduced the inhibitory effect of TRIM13 overexpression on LC cell proliferation and metastasis

**FIGURE 4:**
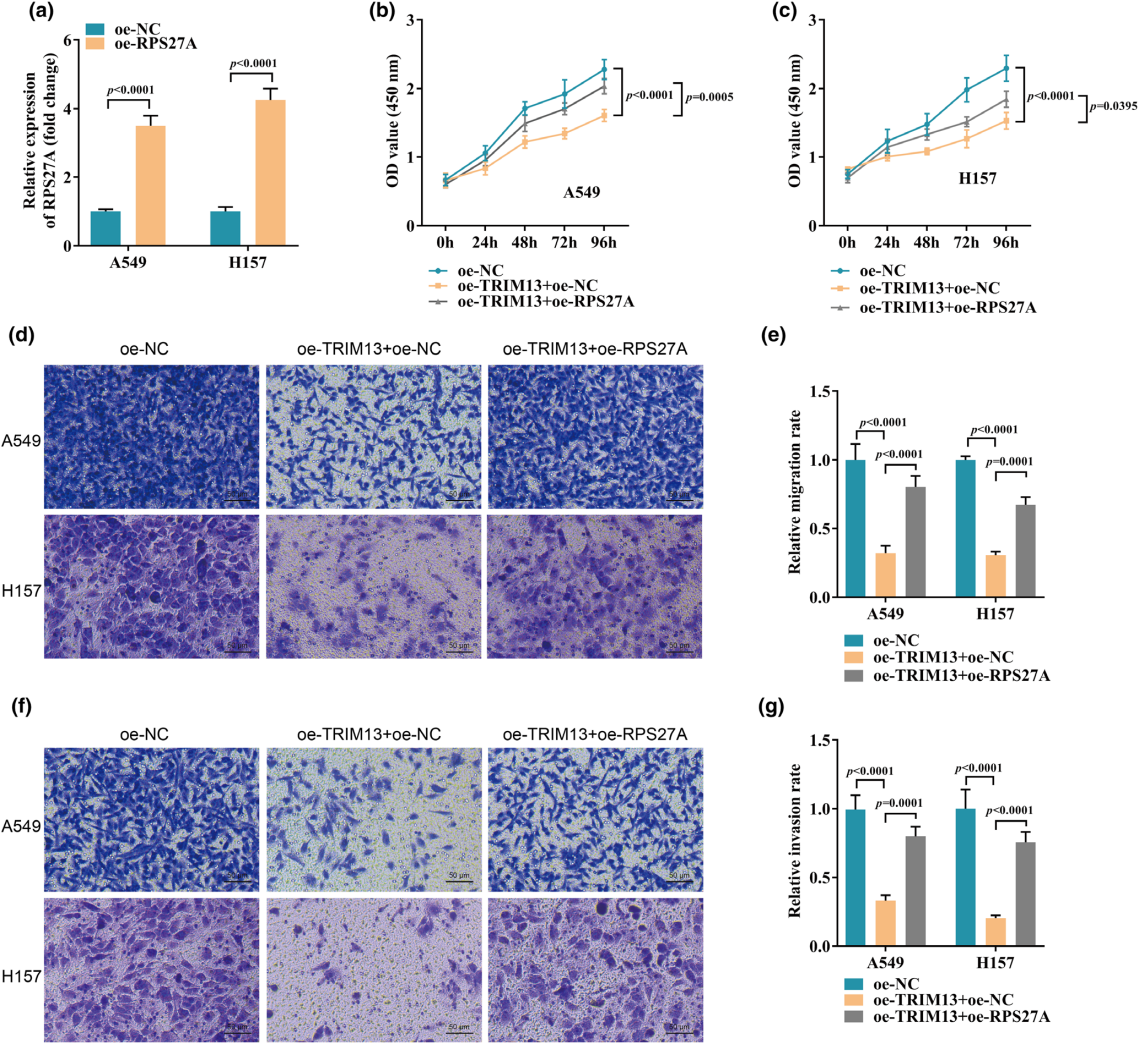
Overexpression of RPS27A significantly reduced the inhibitory effect of TRIM13 overexpression on LC cell proliferation and metastasis. (a) The transfection efficiency of RPS27A in LC cells; (b, c) Cell proliferation was evaluated by CCK‐8 assay; (d–g) Cell migration (d, e) and invasion (f, g) were detected by the transwell assay.

Next, we hypothesized that TRIM13 might act as a tumor suppressor gene by ubiquitinating and degrading RPS27A, ultimately attenuating the proliferation and metastasis of LC cells. To verify this hypothesis, we upregulated the expression level of RPS27A in LC cells by cell transfection and checked the transfection efficiency by qRT‐PCR. The results showed that oe‐RPS27A transfection significantly up‐regulated the expression level of RPS27A in A549 and H157 (Figure 4a). Subsequently, we examined the effect of RPS27A overexpression on tumor cell biological functions by CCK‐8 and transwell assays. The results showed that overexpression of RPS27A significantly reduced the inhibitory effects of TRIM13 overexpression on A549 and H157 cell proliferation (Figure 4b,c), migration (Figure 4d,e) and invasion (Figure 4f,g).

### 3.5 NF‐kB signaling pathway participates in the regulation of TRIM13/RPS27A axis on LC cell proliferation, invasion, and migration

**FIGURE 5:**
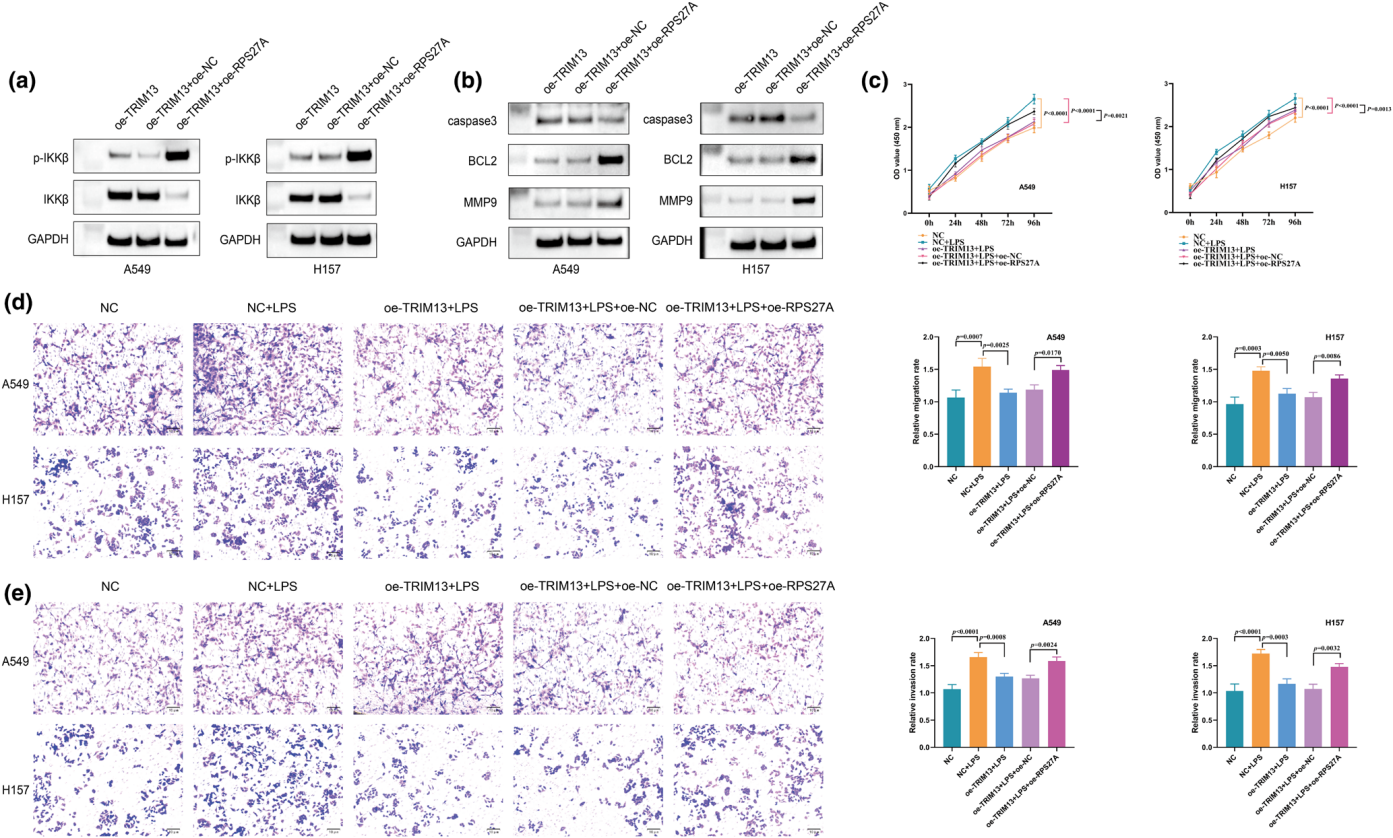
NF‐kB signaling pathway participates in the regulation of TRIM13/RPS27A axis on LC cell proliferation, invasion and migration. (a, b) The expression levels of IKKβ and phosphorylated IKKβ (a) and NF‐kB downstream effectors including MMP9, BCL2, and caspase 3 (b) in LC cells transfected with oe‐TRIM13 and oe‐RPS27A were evaluated by western blot; (c) Cell proliferation was evaluated by CCK‐8 assay; (d, e) Cell migration (d) and invasion (e) were detected by the transwell assay.

The aforementioned studies have shown that TRIM13 overexpression inhibits the activation of the NF‐κB pathway in LC (Xu et al., 2019). In order to explore whether TRIM13 can regulate the activation of NF‐κB pathway in LC through RPS27A, we detected the protein level of IKKβ (IKB kinase β) and the expression of phosphorylated IKKβ (p‐IKKβ) by western blot. Compared with A549 and H157 cells overexpressing TRIM13, decreased IKKβ levels and increased p‐IKKβ levels were found in A549 and H157 cells overexpressing TRIM13 and RPS27A (Figure 5a). In addition, we evaluated the protein expression of NF‐κB downstream effectors including MMP9, BCL2, and caspase 3. Overexpression of RPS27A upregulated the protein levels of MMP9 and BCl2 and downregulated the expression of caspase‐3 in A549 and H157 cells (Figure 5b). The above study results indicated that RPS27A overexpression reversed the inhibitory effect of TRIM13 upregulation on the activation of NF‐kB signaling pathway in LC. In order to further explore whether the NF‐κB pathway was involved in the regulation of TRIM13/RPS27a on LC cell proliferation, migration and invasion, we conducted cell function experiments. CCK‐8 results showed that after adding LPS to TRIM13‐overexpressing A549 and H157 cells, the OD values after co‐incubation at 24, 48, 72, and 96 h were reduced. However, the additional RPS27A increased the OD value in TRIM13‐overexpressing A549 and H157 cells (Figure 5c). This suggested that the activation of NF‐kB was related to the regulation of cell proliferation by TRIM13/RPS27A. Transwell analysis showed that oe‐TRIM13 transfection of A549 and H157 cells could reverse the upregulation of cell invasion and migration caused by LPS. Co‐transfection of TRIM13 and RPS27A overexpression plasmids into A549 and H157 cells supplemented with LPS reversed the downregulation of cell invasion and migration mediated by overexpression of TRIM13 (Figure 5d,e). This suggested that the activation of the NF‐kB pathway was closely related to the regulation of cell invasion and migration by TRIM13/RPS27A.

### 3.6 TRIM13 suppressed LC tumor growth by interacting with RPS27A in vivo

**FIGURE 6:**
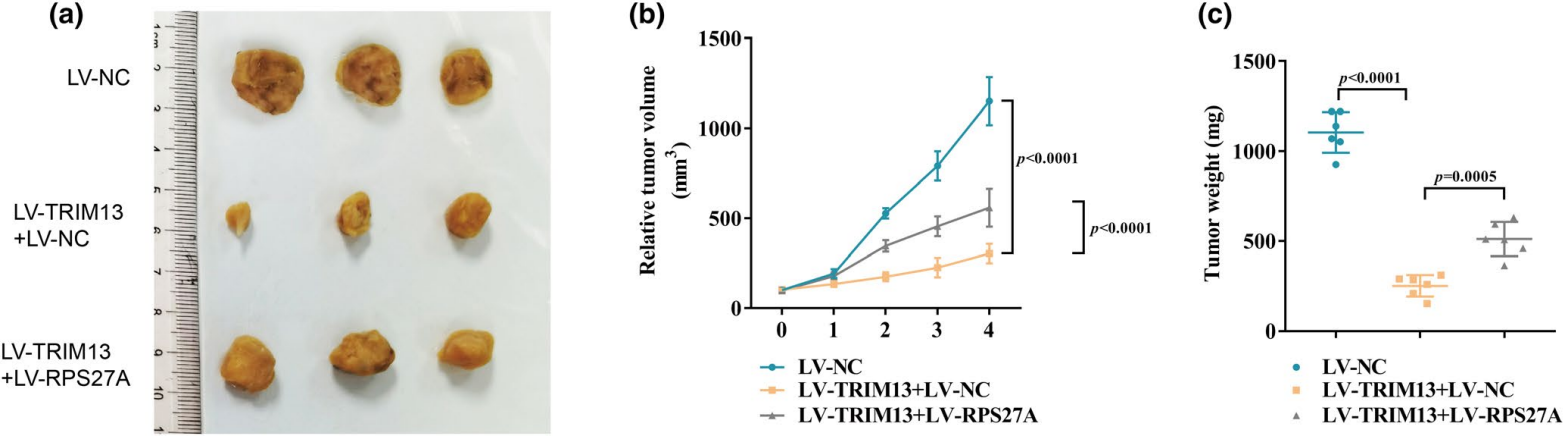
TRIM13 suppressed LC tumor growth by interacting with RPS27A in vivo. (a) Tumor image was photoed 4 weeks later; (b) Tumor volume was monitored every week; (c) Tumor weight was measured 4 weeks later.

To explore the regulatory role of TRIM13‐RPS27A interaction on LC progression in vivo, we constructed a LC xenograft mouse model by subcutaneously injecting A549 cells into BALB/c nude mice. One week after inoculation, mice were treated weekly with control adenovirus, TRIM13‐overexpressing adenovirus and RPS27A‐overexpressing adenovirus via tail vein injection. We found that TRIM13 overexpression significantly inhibited the increase in tumor volume (Figure 6a,b) and weight (Figure 6c) in mice compared to the LV‐NC group. In addition, the inhibitory effects of TRIM13 overexpression on tumor volume (Figure 6a,b) and weight (Figure 6c) were partially blocked by RPS27A overexpression. The above results suggested that TRIM13 inhibited LC progression by interacting with RPS27A in vivo.

## 4 DISCUSSION

Accumulated research evidence shows that TRIM proteins can act as oncogenes or tumor suppressor genes to regulate the progression of various tumors (Giannopoulou et al., 2022; Ma et al., 2016; Yang et al., 2018). For example, in the hypoxic microenvironment of tumors, high expression of TRIM14 in colorectal cancer (CRC) significantly suppresses CRC cell apoptosis and promotes CRC cell proliferation and invasion (Jing et al., 2022). In another study, TRIM15 has been confirmed to enhance non‐small cell LC progression by increasing Nrf2 stability mediated through Keap1 ubiquitination and degradation (Liang et al., 2022). Simultaneously, some TRIM proteins may exhibit opposite effects. For instance, TRIM22 has been shown to inhibit osteosarcoma progression by activating the ROS/AMPK/mTOR/autophagy signaling pathway through weakening Nrf2 protein stability (Liu et al., 2022). In addition, recent studies have also shown that some abnormally expressed TRIM proteins are closely related to the diagnosis and prognosis of malignant tumors (Dai et al., 2021; Zhang et al., 2018). For example, Xiao et al. conducted a comprehensive search of the Web of Science, PubMed, CNKI, Wanfang, EMBASE, and Google Scholar databases to systematically evaluate the prognostic value of TRIM44 in malignant tumor patients. The analysis results revealed that overexpression of TRIM44 protein was significantly associated with shorter OS and poorer disease‐free survival (DFS) in malignant tumor patients. Additionally, increased levels of TRIM44 protein expression were correlated with lymph node metastasis, distant metastasis, increased tumor infiltration depth, advanced clinical stage, and recurrence. TRIM44 may serve as a valuable prognostic biomarker and potential therapeutic target for malignant tumor patients (Xiao et al., 2020). This study confirmed by western blot and IHC analysis that TRIM13 was significantly underexpressed in LC tumor tissues and cell lines. Moreover, compared with patients with high TRIM13 expression, patients with low TRIM13 expression had significantly shorter OS. Furthermore, upregulation of TRIM13 significantly inhibited the proliferation and metastasis of LC cells in vitro, and attenuated tumor growth in mice in vivo. The above findings consistently indicated that low expression of TRIM13 may act as a tumor suppressor gene to inhibit the development and metastasis of LC.

TRIM proteins are often involved in cancer progression through ubiquitination and degradation of target proteins (Bertrand et al., 2008). However, the underlying molecular mechanism of TRIM13 in LC progression are still uncertain. In this study, 10 target proteins that may interact with TRIM13 to participate in the regulation of LC progression were screened out through the GEO database. Subsequently, the interaction between TRIM13 and the target protein RPS27A in LC cells was confirmed by western blot and Co‐IP experiments. RPS27A is a member of the ribosomal protein S27AE family and one of the protein components of the ribosomal 40S small subunit (Redman & Rechsteiner, 1989). In addition to participating in protein synthesis, ribosomal proteins also have other important functions outside ribosomes, such as transcription processing, repair, autotranslation, regulation of apoptosis, and regulation of malignant transformation and development of normal cells (Warner & McIntosh, 2009; Wool, 1996). Recent studies have shown that RPS27A protein is abnormally expressed in colon cancer, kidney cancer, breast cancer and LUAD, and is associated with tumorigenesis (Adams et al., 1992; Kanayama et al., 1991; Wong et al., 1993). Wang et al. found that high expression of RPS27A in cervical cancer is associated with poor prognosis in patients with advanced cervical cancer, and acts as an oncogene for HPV16 cervical cancer development to promote tumor progression (Wang et al., 2021). Li et al. showed that downregulation of RPS27A expression induces cell cycle arrest and apoptosis in LUAD cells and plays an important role in LUAD progression and prognosis (Li, Zhang, et al., 2022). In addition, Mu et al. found that RPS27A expression was downregulated in CRC, and its overexpression promoted CRC cell growth and inhibited cell apoptosis (Mu et al., 2021). An increasing number of studies have shown that RPS27A is involved in regulating the progression of multiple cancer types. However, the role of RPS27A in LC remains to be investigated.

In this study, we elucidated the specific molecular mechanism by which TRIM13 regulates LC progression and metastasis. TRIM13 bound to the target protein RPS27A and promoted the ubiquitination and degradation of RPS27A. This was supported by in vivo and in vitro experimental evidence. In the first place, TRIM13 overexpression reduced the expression level of RPS27A in LC cells. Consequently, the results of Co‐IP assay showed that TRIM13 can be precipitated by RPS27A. Furthermore, upregulation of TRIM13 resulted in a marked increase in the amount of ubiquitin co‐immunoprecipitated with RPS27A. TRIM13 overexpression promoted RPS27A downregulation induced by the proteasome inhibitor CHX in LC cells. Cell function experiments revealed that RPS27A overexpression partially reversed the inhibitory effect of TRIM13 overexpression on LC cell proliferation and metastasis. Finally, in vivo experiments revealed that RPS27A overexpression partially blocked the inhibitory effect of TRIM13 overexpression on tumor growth in LC xenograft mice. Taking the above findings together, we confirmed that TRIM13 overexpression inhibited LC progression in vivo and in vitro by promoting the ubiquitination and degradation of RPS27A.

The NF‐κB transcription factor family is activated under various intracellular or extracellular stimuli, and its dysregulation can lead to pathological conditions such as infection, cancer, and neurodegenerative diseases. Existing studies have shown that TRIM family proteins play a key role in the regulation of the NF‐kB pathway in various cancers (Fan et al., 2017; Tomar & Singh, 2015). Dhanendra Tomar et al. found that TRIM13 inhibited the activation of the NF‐κB pathway in LC cells by regulating the ubiquitination of NEMO (Tomar & Singh, 2014). Xu et al. have confirmed that TRIM13 inhibits the proliferation and metastasis of non‐small cell LC cells by mediating the inactivation of the NF‐κB pathway (Xu et al., 2019). To determine whether TRIM13 can regulate the NF‐kB pathway in LC by regulating RPS27A, we observed the activity of the NF‐kB signaling pathway. In LC cell lines, RPS27A overexpression reversed the inhibitory effect of TRIM13 overexpression on NF‐kB activation. These results indicated that TRIM13/RPS27A was a potential regulator of the NF‐kB pathway in LC. To further explore whether the NF‐kB signaling pathway was involved in the regulation of TRIM13/RPS27A on the occurrence and development of LC, the function of LC cells was examined. The results showed that TRIM13 overexpression could reverse the LPS‐mediated upregulation of cell invasion, migration, and proliferation. In contrast, RPS27A overexpression alleviated the TRIM13‐mediated inhibitory effect on LPS‐induced upregulation of cell invasion, migration, and proliferation. The above results strongly suggested that LPS‐induced activation of NF‐kB affected the TRIM13/RPS27A‐mediated regulation of LC development.

## 5 CONCLUSION

Taken together, this study firstly demonstrated that TRIM13 inhibited LC progression by interacting with RPS27A and promoting ubiquitination and degradation. In addition, our findings strongly supported that TRIM13 was involved in regulating the inactivation of the NF‐kB pathway in LC by regulating RPS27A. The NF‐kB pathway took a part in the regulation of TRIM13/RPS27A‐mediated LC development. Therefore, targeting the TRIM13/RPS27A/NF‐kB axis may be a promising target for the treatment of LC.

## AUTHOR CONTRIBUTIONS

**Lailing Li** and **Hui Zhou:** Conceptualization, validation, writing–original draft. **Yayun Cui:** Formal analysis, investigation, visualization. **Ke Xu:** Conceptualization, supervision, writing‐review and editing. All authors reviewed the results and approved the final version of the manuscript.

## FUNDING INFORMATION

This study was supported by Anhui Provincial Cancer Hospital Youth Foundation (2023YJQN004).

## CONFLICT OF INTEREST STATEMENT

The authors declare that they have no known competing financial interests or personal relationships that could have appeared to influence the work reported in this paper.

## ETHICS STATEMENT

This study was approved by the Ethics Committee of The First Affiliated Hospital of USTC, Division of Life Sciences and Medicine, University of Science and Technology of China (Anhui Provincial Cancer Hospital). All participants were provided with written informed consent at the time of recruitment, and all experiments involving human tissue specimens comply with the *Declaration of Helsinki*. Animal studies were performed in compliance with the ARRIVE guidelines.

## Data Availability

The data that support the findings of this study are available from the corresponding author upon reasonable request.
